# Many, more, most: four risk profiles of adolescents in residential care with major psychiatric problems

**DOI:** 10.1186/s13034-017-0204-1

**Published:** 2017-12-19

**Authors:** Elisabeth A. W. Janssen-de Ruijter, Eva A. Mulder, Jeroen K. Vermunt, Chijs van Nieuwenhuizen

**Affiliations:** 1 GGzE Centre for Child & Adolescent Psychiatry, PO BOX 909 (DP 8001), 5600 AX Eindhoven, The Netherlands; 2 Scientific Center for Care & Welfare (Tranzo), Tilburg University, Tilburg, The Netherlands; 3 Leiden University Medical Center, Leiden, The Netherlands; 4 Intermetzo-Pluryn, Nijmegen, The Netherlands; 5 Department of Methodology and Statistics, Tilburg University, Tilburg, The Netherlands

**Keywords:** Disruptive behaviour, Risk factors, Latent class analysis, Forensic psychiatry

## Abstract

**Background:**

The development of delinquent behaviour is largely determined by the presence of (multiple) risk factors. It is essential to focus on the patterns of co-occurring risk factors in different subgroups in order to better understand disruptive behaviour.

**Aims and hypothesis:**

The aim of this study was to examine whether subgroups could be identified to obtain more insight into the patterns of co-occurring risk factors in a population of adolescents in residential care. Based on the results of prior studies, at least one subgroup with many risk factors in multiple domains and one subgroup with primarily risk factors in a single domain were expected.

**Methods:**

The structured assessment of violence risk in youth and the juvenile forensic profile were used to operationalize eleven risk factors in four domains: individual, family, peer and school. Data from 270 male adolescents admitted to a hospital for youth forensic psychiatry and orthopsychiatry in the Netherlands were available. Latent class analysis was used to identify subgroups and significant differences between the subgroups were examined in more detail.

**Results:**

Based on the fit statistics and the clinical interpretability, the four-class model was chosen. The four classes had different patterns of co-occurring risk factors, and differed in the included external variables such as psychopathology and criminal behaviour.

**Conclusions:**

Two groups were found with many risk factors in multiple domains and two groups with fewer (but still several) risk factors in single domains. This study shed light on the complexity of disruptive behaviour, providing a better insight into the patterns of co-occurring risk factors in a heterogeneous population of adolescents with major psychiatric problems admitted to residential care.

**Electronic supplementary material:**

The online version of this article (10.1186/s13034-017-0204-1) contains supplementary material, which is available to authorized users.

## Background

The development and persistence of delinquent behaviour in youth is largely determined by the presence of (multiple) risk factors. Most research in youth forensic psychiatry has focused on which risk factors predict delinquency and how (persistent) delinquent behaviour in youth can be prevented [1–3]. These studies suggest that interventions that focus on delinquency must be aimed at reducing risk factors, in line with the risk-need-responsivity model (RNR-model) of Andrews and Bonta [4]. This model describes that the intensity of treatment should be adjusted to the nature, extent and severity of the problems. In addition to the nature, extent and severity of the risk factors, insight into the patterns of co-occurring risk factors is relevant to the treatment of this high-risk youth, because the interaction of multiple risk factors may influence treatment outcomes. Furthermore, studying the co-occurrence of risk factors in youth with major psychiatric problems manifesting behavioural maladjustment, could gain more insight into the complexity of disruptive and delinquent behaviour.

In many studies on the development of delinquent behaviour, risk factors are divided into different domains: the individual, family, peer and school domains [2, 3, 5]. Examples of risk factors for delinquency are low IQ and prior history of substance use in the individual domain [3, 5, 6], exposure to violence in the home and parental criminality in the family domain [2, 3, 5, 7, 8], peer rejection and delinquent peers in the peer domain [3, 5, 6, 9] and low academic achievement and truancy in the school domain [2, 3, 5, 9]. Many adolescents with delinquent behaviour have multiple risk factors in numerous domains in their lives [9].

Possible consequences of being exposed to multiple risk factors have been described in the cumulative risk hypothesis [10, 11]. This hypothesis implies that the accumulation of risk factors, regardless of the presence or absence of particular risk factors, affects developmental outcomes: the greater the number of risk factors, the greater the prevalence of delinquent behaviour. Several studies have confirmed such a dose–response relationship between the number of risk factors and the likelihood of delinquent behaviour [2, 3, 5, 6, 9, 12]. Furthermore, exposure to an accumulation of risk factors in multiple domains, instead of risk factors in a single domain, increases the chance of later negative outcomes such as delinquent behaviour [12].

Despite the substantial number of studies on (multiple) risk factors for delinquent behaviour, little is known about the patterns of co-occurring risk factors among adolescents. To study the co-occurrence of risk factors, a person-centred approach instead of a variable-centred approach is needed. A person-centred approach examines how behaviours co-occur in groups of adolescents. In most research with a person-centred approach, subgroups are based on specific characteristics, such as committed offences, emotional and behavioural problems, or one single risk factor such as substance abuse [13–17]. In addition, the studies that used multiple risk factors to find subgroups have examined specific populations, such as childhood arrestees or first offenders [18–20]. However, studies on subgroups based on multiple risk factors in a broad population of adolescents in residential care are scarce.

Adolescents in residential care are a heterogeneous population, for example concerning psychiatric problems and exposure to risk factors [21, 22]. In addition, disruptive problem behaviour and delinquent behaviour are quite common in this population, although the frequency and severity of these behaviours may differ [23]. Insight into the patterns of co-occurring risk factors is a first step to better understanding the complexity of disruptive behaviour. Therefore, the aim of this study was to examine whether subgroups could be identified to obtain more insight into the patterns of co-occurring risk factors in a heterogeneous population of adolescents in residential care with no, minor or serious delinquent behaviour and major psychiatric problems. Based on the results of prior studies on multiple risk factors, at least one subgroup with many risk factors in multiple domains and one subgroup with primarily risk factors in a single domain were expected [18, 19].

## Methods

### Setting

All participants were admitted to the Catamaran, a hospital for youth forensic psychiatry and orthopsychiatry in the Netherlands. This secure residential care setting offers intensive multidisciplinary treatment to male and female patients aged between 14 and 23 years. Patients admitted to this hospital are sentenced under juvenile criminal law or juvenile civil law, or are admitted voluntarily. Dutch juvenile criminal law comprises the treatment and rehabilitation of adolescents^1^ who have committed serious offences. Measures under the Dutch juvenile civil law are applied to adolescents whose development is at risk and whose parents or caregivers are not able to provide the required care. Irrespective of the type of measure, all patients of this hospital display severe and multiple problems in different areas of their lives.

### Participants

The total sample comprised all male patients admitted to the Catamaran with a minimal stay of 3 months between January 2005 and July 2014 (N = 275). Because 99% of the admitted adolescents are male, only male patients were included. Five patients who objected to the provision of the data for research purposes were excluded from the sample. Hence, the final sample comprised 270 patients. Of these patients, 129 were sentenced under Dutch juvenile criminal law (47.8%) and 118 under Dutch juvenile civil law (43.7%), while 23 patients were admitted voluntarily (8.5%). The majority of the patients (81.1%) were convicted of one or more offence(s) before their admission. Moderately violent offences (50.0%) and property offences without violence (45.2%) were the most common. As for psychopathology, most of the DSM-IV-TR disorders were in the category “disorders usually first diagnosed in infancy, childhood, or adolescence”, in particular disruptive behaviour disorders (48.9%) and autism spectrum disorders (42.6%). Detailed demographic characteristics are displayed in Table 1.

**Table 1:** Demographic characteristics (N = 270)

	M (SD)	Range
Age at admission in years	16.9 (1.8)	14–23
IQ	93.9 (12.0)	63–127

	*n*	%
Judicial measure		
Criminal law	129	47.8
Civil law	118	43.7
Voluntary	23	8.5
Previous delinquent behaviour^a^		
No conviction	51	18.9
Drug offence	12	4.4
Vandalism (property)	83	30.7
Property offence without violence	122	45.2
Moderate violent offence	135	50.0
Violent property offence	53	19.6
Serious violent offence	21	7.8
Sex offence	36	13.3
Manslaughter	9	3.3
Arson	2	0.7
Murder	7	2.6
Axis-I classification of DSM-IV-TR^b,c^		
Disruptive behaviour disorder	132	48.9
Autism spectrum disorder	115	42.6
Attention deficit/hyperactivity disorder	63	23.3
Substance disorder	61	22.6
Reactive attachment disorder	34	12.6
Schizophrenia or other psychotic disorder	25	9.3
Mood disorder	23	8.5
Anxiety disorder	22	8.1
Other disorder usually first diagnosed in infancy, childhood, or adolescence	19	7.0
Other disorders^d^	18	6.7
Axis-II classification of DSM-IV-TR^b^		
Personality disorder	16	5.9
Mental retardation	16	5.9

^a^Classification of Van Kordelaar [28]

^b^Only DSM-IV-TR classifications with a prevalence of > 5% are displayed

^c^Due to comorbidity, percentages of DSM-IV-TR classifications do not sum up to 100

^d^Other disorders are sexual and gender identity disorders, sleep disorders, impulse control disorders not elsewhere classified, and adjustment disorders

### Data collection

Data were collected through the structured assessment of violence risk in youth, the juvenile forensic profile and structured file analysis.

#### Structured assessment of violence risk in youth (SAVRY)

The SAVRY [24] is a risk assessment tool based on the structured professional judgement model. The SAVRY consists of 24 risk items and six protective items. The risk items have three coding possibilities (low, moderate and high), whereas the protective items are scored on a two-point scale (present or absent). The inter-rater reliability of the SAVRY risk total score is good and the predictive validity for physical violence against persons is excellent [24, 25].

#### Juvenile forensic profile (JFP)

The JFP [26] has been developed to measure risk factors in all life areas and for all types of offending behaviour using file data. The instrument contains seventy risk factors pertaining to seven domains: history of criminal behaviour, family and environment, offence-related risk factors and substance use, psychological factors, psychopathology, social behaviour/interpersonal relationships, and behaviour during stay at the institution. Each risk factor is measured on a three-point scale, where 0 = no problems, 1 = some problems, and 2 = severe problems. The inter-rater reliability of the JFP and the convergent validity, measured by SAVRY, were of satisfactory quality [26]. The predictive validity of the JFP was tested in a sample of 102 boys. A total score from nine risk factors of the JFP was found to be a good predictor of recidivism (AUC of 0.80; [27]).

#### Structured file analysis

Structured file analysis was used to register objective characteristics of the patients’ lives. These characteristics included general background information (for example, ethnicity), life events, DSM-IV-TR classifications and committed offences. The committed offences were classified in accordance with the classification by Van Kordelaar ([28]; as used in [17]) and the life events were based on the ‘Life Events’ scoring list from a Dutch monitor system for youth health [29].

### Data preparation

In this study, risk factors that were present at the moment of admission to the hospital were used to identify distinct subgroups. Therefore, eleven risk factors within the four domains (individual, family, peer and school), which were often described in the literature as prominent risk factors for disruptive problem behaviour or delinquency, were chosen. The best appropriate items of the SAVRY and JFP were used to operationalize these eleven risk factors.

The individual domain consisted of three risk factors: hyperactivity (item 43 of the JFP), cognitive impairment (item 39 of the JFP) and history of drug abuse (item 42 of the JFP). The family domain contained three risk factors: exposure to violence in the home (item 6 of the SAVRY), childhood history of maltreatment (item 7 of the SAVRY) and criminal behaviour of family members (item 14 of the JFP). The three risk factors in the peer domain were peer rejection (item 10 of the JFP), involvement in criminal environment (item 13 of the JFP) and lack of secondary network (item 55b of the JFP). The school domain comprised two risk factors: low academic achievement (item 25 of the JFP) and truancy (item 22 of the JFP).

After the identification of the different subgroups, possible differences between the subgroups were examined. For this, the objective characteristics from the file analysis and two age variables of the JFP (age of first criminal behaviour/violent behaviour) were used.

### Procedure

Scoring of the SAVRY and JFP was done by officially trained and certified researchers and trainees under supervision. All instruments were completed by means of consensus scoring until an inter-rater reliability of at least 80% was achieved. After reaching an inter-rater reliability of at least 80%, the certified researchers scored individually. The trainees who were not officially trained remained under the supervision of a trained researcher, which means that each SAVRY and JFP they scored was checked by a trained researcher. The procedure scoring the structured file analysis was identical: after achieving an inter-rater reliability of at least 80%, the researchers scored individually and the trainees remained under the supervision of a researcher.

Scoring of the historical items of the SAVRY and JFP and the structured file analysis took place simultaneously 3 months after admission of the patient. At that time, all the required documents had been collected and the patient files were (mostly) complete. Risk factors, life events and other variables before admission were scored using information from all possible sources before admission, such as diagnostic reports from psychologists and psychiatrists, criminal records, treatment plans from previous settings and juridical documents. DSM-IV-TR classifications, demographic information and admission characteristics were collected from registration files and the first treatment plan of the Catamaran. All information was processed anonymously.

The Dutch Law on Medical Treatment Agreement Article 7: 458 states that scientific research is permitted without the consent of the patient if an active informed consent is not reasonably possible or, given the type and aim of the study, may not be required. The anonymity of the patient must be ensured using coded data. In addition, scientific research without the active consent of the patient is only permitted under three conditions: (1) the study is of general interest; (2) the study cannot be conducted without the requested information; and (3) the participant has not expressly objected to the provision of the data. This study fits within the conditions of this law, as the data were collected retrospectively. For an extra check, this type of study has been discussed thoroughly and approved by the science committee of the GGzE and by the Ethics Review Board of Tilburg University. In this study, patients’ anonymity was guaranteed by using research numbers instead of names. Five patients in the initial sample (N = 275) explicitly objected to the provision of the data for research purposes and were therefore excluded. Hence, this study was conducted in accordance with the prevailing medical ethics in the Netherlands.

### Statistical analyses

Latent class analysis (LCA) by means of Latent GOLD 5.0 [30, 31] was used to construct a clustering of latent classes based on a set of categorical latent variables [32]. In LCA, the following three steps were used: (1) a latent class model was built using the eleven risk factors as indicators; (2) subjects were assigned to latent classes based on their posterior class membership probabilities; and (3) the relationship between class membership and external variables was investigated [33].

In the first step, a latent class model was built with eleven ordinal risk factors as indicators. Of these factors, ten risk factors used a three-point scale: 0 (no risk), 1 (a small risk) and 2 (a high risk), and the eleventh risk factor (cognitive impairment) was recoded into a dichotomous variable (IQ less than or equal to 85 versus higher than 85). To identify the most suitable number of classes, several model fit indices were used. Firstly, the complexity of the latent class model was considered using three information criteria: the Bayesian information criterion (BIC), the Aikake information criterion (AIC) and the Aikake information criterion 3 (AIC3; [32, 34–37]). These criteria weight the fit and the parsimony of a model: the criteria are lowest for the best model. Secondly, a bootstrap likelihood ratio test (BLRT; [38]) was used to compare two models—for example, the three-class model with the four-class model. A significant p value (*p* < .05) rejects the null hypothesis that the three-class model, in this example, holds in the population.

In step two, the subjects were assigned to latent classes based on their posterior class membership probabilities. The classification method was a proportional assignment, which means that subjects were assigned to each class with a weight equal to the posterior membership probability for that class [32].

In the last step (step three), the association between class membership and external variables was investigated. For this purpose, the BCH method for continuous data [39] and the maximum likelihood (ML) procedure for nominal data [40] were used. Wald tests were used to determine the significance (*p* < .05) of the encountered differences between classes in external variables (e.g. life events and committed offences). The significance tests are mainly used to eliminate the variables which are of less interest rather than to prove which effects really exist. Therefore, the alpha level is not adjusted for multiple testing (e.g. using a Bonferroni correction of a factor 53) since much stricter alpha levels would potentially hide possibly interesting correlates of the encountered classes.

## Results

### LCA

Table 2 shows the model fit statistics for models between one and eight latent classes. For the optimal modelling of the data, the information criteria suggest a range of a three-class model (BIC) to a seven-class model (AIC). The AIC3, which is the suitable criterion to use in small samples [34], is lowest for the four-class model. The *p* values of the BLRT were significant up to and including the four-class model. This means that the four-class model was preferred over the three-class model (BLRT = 44.44, *p* < .000). Therefore, the four-class solution was chosen, which was also in line with the clinical interpretability of the classes.

**Table 2:** Model fit statistics for latent classes

	LL	BIC	AIC	AIC3	No. of para-meters	*p* value BLRT	Entropy *R* ^*2*^
1-class	− 2444.22	5006.02	4930.45	4951.45	21		1.00
2-class	− 2396.34	4977.42	4858.67	4891.67	33	.000	.67
3-class	− 2359.75	4971.42	4809.49	4854.49	45	.000	.68
4-class	− 2337.52	4994.16	4789.05	4846.05	57	.000	.71
5-class	− 2322.49	5031.28	4782.99	4851.99	69	.064	.73
6-class	− 2308.20	5069.88	4778.41	4859.41	81	.168	.73
7-class	− 2294.16	5108.97	4774.32	4867.32	93	.116	.75
8-class	− 2282.86	5153.56	4775.72	4880.72	105	.296	.76

*LL* log likelihood, *BIC* Bayesian information criterion, *AIC* Aikake information criterion, *AIC3* Aikake information criterion 3, *BLRT* bootstrap likelihood ratio test

### Class description

**Fig. 1:**
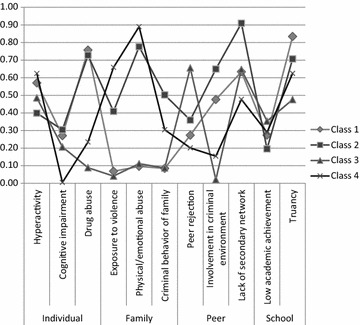
Four-class class solution (N = 270)

The means of the risk factors in the individual, family, peer and school domains for each of the four classes on a zero to one scale are shown in Fig. 1. Table 3 shows significant differences between the four classes on all risk factors except for hyperactivity, cognitive impairment and low academic achievement. Class 1 (*n* = 119, 44% of sample) represented adolescents with risk factors in the individual domain (drug abuse), peer domain (involvement in criminal environment) and school domain (truancy). In addition, adolescents in Class 2 (*n* = 70, 26% of sample) had risk factors in all four domains, such as drug abuse, childhood history of maltreatment and lack of a secondary network. In contrast, adolescents in Class 3 (*n* = 49, 18% of sample) had the lowest risks overall. Notably, they had the highest risk for peer rejection compared to the adolescents in other classes. Finally, Class 4 (*n* = 32) represented the smallest group of adolescents (12% of sample). Risk factors that were common in this group were exposure to violence in the home and childhood history of maltreatment in the family domain.

**Table 3:** Means and comparison of LCA variables across four classes (N = 270)

Risk factors	Overall mean	Class 1 (*n* = 119)	Class 2 (*n* = 70)	Class 3 (*n* = 49)	Class 4 (*n* = 32)	Wald	*p*	Post hoc
Hyperactivity	1.03	1.14	.80	.97	1.25	5.59	.140	–
Cognitive impairment	.24	.27	.30	.21	.01	1.79	.620	–
History of drug abuse	1.12	1.51	1.46	.18	.47	26.88	.000	1,2 > 3,4
Exposure to violence in the home	.43	.14	.82	.08	1.32	26.01	.000	2,4 > 1; 4 > 3
Childhood history of maltreatment	.74	.19	1.55	.22	1.78	14.06	.003	2,4 > 1,3
Criminal behaviour of family members	.44	.17	1.00	.17	.61	21.47	.000	2,4 > 1; 2 > 3
Peer rejection	.72	.55	.72	1.31	.40	16.40	.001	3 > 1,2,4
Involvement in criminal environment	.78	.95	1.30	.04	.31	23.76	.000	1,2 > 3,4; 2 > 1
Lack of secondary network	1.38	1.27	1.82	1.30	.95	13.01	.005	2 > 1,3,4
Low academic achievement	.54	.55	.39	.71	.58	31.9	.36	–
Truancy	1.42	1.67	1.41	.95	1.25	15.81	.001	1,2 > 3; 1 > 4

### Profiling the classes

To further describe the four classes, differences between the classes concerning the demographic and admission characteristics, psychopathology, drug use, criminal behaviour and life events were studied (see Additional file 1). The following variables were significantly different between the classes: judicial measure, age at admission, ethnicity and earliest age of (outpatient) care. More specifically, there were more first and second generation immigrants in Class 2 than in Classes 1 and 3 (Wald = 13.70, *p* = .003). The majority of adolescents in Class 2 were placed under the Dutch juvenile criminal law, whereas the majority of adolescents in Class 4 were placed under the Dutch civil law (Wald = 16.09, *p* = .013). In addition, adolescents in Class 4 had the earliest age of (outpatient) care (mean = 6.8; Wald = 8.33, *p* = .040) and were youngest at admission to the Catamaran (mean = 15.6; Wald = 24.44, *p* = .000).

As for psychopathology, the following disorders differed significantly between the classes: disruptive behaviour disorder, autism spectrum disorder, substance disorder, reactive attachment disorder and schizophrenia or other psychotic disorder. Adolescents in Classes 1 and 2 were, compared to adolescents in Classes 3 and 4, more often diagnosed with a disruptive behaviour disorder (Wald = 11.37, *p* = .010), a substance disorder (Wald = 194.67, *p* = .000), and schizophrenia or other psychotic disorder (Wald = 103.47, *p* = .000). Furthermore, autism spectrum disorders were more common in adolescents in Classes 1 and 3 (Wald = 28.64, *p* = .000), and reactive attachment disorders were more common in adolescents in Classes 2 and 4 (Wald = 15.83, *p* = .001). In addition, substance use differed significantly between the classes—soft drug use (Wald = 49.64, *p* = .000), hard drug use (Wald = 214.33, *p* = .000) and alcohol use (Wald = 41.83, *p* = .000)—and was more common in adolescents in Classes 1 and 2.

With regard to criminal behaviour, there were significant differences in no previous offences, vandalism, property offences without violence, moderate violent offences, violent property offences, serious violent offences, sex offences, arson and murder. Most types of offence—for example, property offences and violent offences—were more common in adolescents in Classes 1 and 2 than in adolescents in Classes 3 and 4. Sex offences were, however, more common in adolescents in Class 3 (44.1%; Wald = 21.37, *p* = .000). Adolescents in Class 4 most often had no previous offences (53.1%; Wald = 18.03, *p* = .000).

Life events that differed significantly between the classes in the individual domain were victim of discrimination, financial problems, being a refugee from another country and out-of-home placement. For example, out-of-home placement before admission was more common in adolescents in Class 4 (82.4%; Wald = 11.42, *p* = .010). In addition, in the family domain, the following life events were significant: chronic illness or hospitalization of brother/sister, drug abuse parents, psychopathology parents, divorced parents, problems with new parent(s), financial problems parents and deceased brother/sister. Most of these life events in the family were more common in Classes 2 and 4 than in adolescents in Classes 1 and 3. Furthermore, two life events in the peer domain were significant: victim of bullying was most common in adolescents in Class 3 (86.1%; Wald = 18.10, *p* = .000), and impregnated a girl was more common in Classes 2 and 4 (respectively 2.2 and 10.2%; Wald = 19.03, *p* = .000).

### Summary of the classes

Based on the risk factors of the first step of the LCA, two subgroups with many risk factors in multiple domains and two subgroups with fewer risk factors in single domains were found. Firstly, the adolescents in the classes with many risk factors (Classes 1 and 2), were mostly similar in respect of the types of offence they committed, except for the higher number of (attempted) murders in Class 2. In addition, the prevalence of psychopathology and substance use was also similar in both classes, except for the higher prevalence of reactive attachment disorder in Class 2. Alternatively, the main difference between these two classes was the high family risk in Class 2. Other differences were ethnicity (more immigrants in Class 2) and financial problems (higher prevalence in Class 2).

The other two subgroups comprised adolescents with fewer, but still several, risk factors in single domains. The risk factors in these two subgroups were very different: adolescents in Class 3 experienced mainly risks in the peer domain, whereas adolescents in Class 4 experienced mainly family risks. Furthermore, adolescents in these two classes also differed in terms of psychopathology (highest prevalence of autism spectrum disorders in Class 3 versus highest prevalence of reactive attachment disorders in Class 4) and committed offences (the highest prevalence of sex offences in Class 3 versus the highest percentage of no previous conviction in Class 4).

## Discussion

In this study, subgroups were investigated in a sample of adolescents in residential care with no, minor or serious delinquent behaviour and major psychiatric problems. The aim of this study was to obtain more insight into the patterns of co-occurring risk factors in order to better understand disruptive problem behaviour. Four subgroups were identified based on eleven risk factors in the individual, family, peer and school domains: Class 1 with many risk factors in the individual, peer and school domains; Class 2 with many risks in all four domains; Class 3 with mainly risks in the peer domain; and Class 4 with mainly risks in the family domain. These results were largely in line with the hypotheses, identifying not one but two subgroups with many risk factors and also not one but two subgroups with fewer risk factors in single domains.

As for the relationship between class membership and previous delinquent behaviour, this study, like many other studies, supports the cumulative risk hypothesis [10, 11]. Adolescents in the two groups with many risk factors had more often committed multiple offences than adolescents in the other two groups. Adolescents in the two groups with fewer, but still several, risk factors also had a history of delinquent behaviour. However, this behaviour was slightly less frequent than that of adolescents with more risk factors. This finding corresponds with a recent study by Wong et al. [9], who found a linear relationship between the accumulative risk level and delinquency: delinquent boys and girls turned out to have higher risk levels than boys and girls without delinquent behaviour.

Those adolescents in the two groups with many risk factors (Classes 1 and 2) have a similar history of criminal behaviour. The combination of committed offences and experienced risk factors in these two classes corresponds with the characteristics of the subgroup violent property offenders found by Mulder et al. [17]. This subgroup consisted of high-frequency offenders with violent and property offences, highest scores on alcohol abuse and high scores for conduct disorder, involvement with criminal peers, criminal behaviour in the family and truancy. Despite the similarities of the classes with this subgroup of violent property offenders, it is remarkable that the current study distinguished not one but two separate classes with one main difference.

The main difference between Classes 1 and 2 is the high number of family risk factors in Class 2, which is in line with the results of Geluk and colleagues [19]. They found an externalizing intermediate problem group that was characterized by externalizing problems in the individual and peer domains and relatively few parenting problems, and a pervasive high problem group with many problems across all domains. The results of this study on childhood arrestees who committed a first offence under the age of 12 imply that the classification of two separate groups based on the presence or absence of risks in the family domain can also be found in childhood.

Risk factors in the family domain were also seen in adolescents in Class 4 with childhood history of maltreatment as the highest family risk factor. In the literature, an association between maltreatment and later (violent) delinquency was found [41–43]. The pattern that abused children themselves commit violence or delinquent behaviour later in life is described as “the Cycle of Violence” [44, 45]. Bender [46] proposed an extension of this cycle with potential intervening risk factors in order to answer the question of why some maltreated youths become juvenile offenders. She found a potential intervention of two factors for males, namely running away from home and association with deviant peers. The association with deviant peers, which mainly occurred in adolescents in Class 2, could possibly explain why the adolescents in Class 2 were more often involved in criminal behaviour than those in Class 4.

Class 3 is a specific class with distinctive risk factors and characteristics different from the other classes. Adolescents in this class were most often diagnosed with an autism spectrum disorder, had the highest risk for peer rejection, and committed sexual offences more often compared to the other classes. The coincidence of an autism spectrum disorder and peer rejection is in line with the literature, which describes that children with autism spectrum disorders have an increased risk of being victims of bullying [47–49]. In addition, the highest prevalence of sexual offences in this class corresponds with a study by ’t Hart-Kerkhoffs et al. [50] who found higher levels of symptoms of autism spectrum disorder in juvenile suspects of sex offences compared with the non-delinquent population. Furthermore, in a review by Van Wijk et al. [51], a relationship was mentioned between peer relationship problems and sexual offences, both of which were present in this group of adolescents.

Strengths of this study include the use of a reasonably large and complex clinical sample and a sophisticated approach to identifying heterogeneous clusters of youths. Nevertheless, there are also limitations to consider. Firstly, a limitation of this study is the use of file information to gather data. In most cases, the files were complete with corresponding information from various sources. However, in some cases, information from different sources was inconsistent. In these cases, additional information about the patient and/or his parents would have been very useful. Although the structured file analysis and scoring of the SAVRY and JFP was thoroughly conducted with all available information, only 4% of the files were double coded in order to achieve an inter-rater reliability of 80%. However, given the small differences between the raters in the training phase (range 68–88%), we concluded that the individually scored cases were reliable scored. Another limitation to consider is that of the generalizability of the findings. Our sample of male patients was admitted to one hospital for youth forensic psychiatry and orthopsychiatry in the Netherlands, which of course calls into question the generalizability of the findings. However, since the Catamaran offers treatment to a specific group of adolescents with major psychiatric problems from all over the country, this sample might well be representative of the population of adolescents with major psychiatric problems and behavioural problems in the Netherlands.

Despite these limitations, the findings of this study may have implications for practice. The risk, needs, and responsivity principles of the RNR-model [4] are important to take into account. First, according to the risk principle, more intensive treatment should be provided to persons with a risk profile with higher risks (adolescents in Classes 1 and 2) than to persons with a risk profile with lower risks (adolescents in Classes 3 and 4). Second, according to the needs principle, interventions should focus on the criminogenic needs of a person, which can be found in the described risk factors of each subgroup. For example, in adolescents in Classes 2 and 4 with high family risks interventions that strengthen protective factors in the family system could be valuable, because in past research protective factors were found to neutralize risk factors [2, 52]. Third, regarding responsivity, interventions must be adapted to the responsivity of the adolescents, which in this study is provided by information concerning cognitive functioning and low academic achievement in the past. Hence, intervention decisions based on these three principles should finally lead to a reduction of recidivism [4].

In conclusion, this study underscores the importance of person-centred research using multiple risk factors and provides a better insight into the patterns of co-occurring risk factors in a heterogeneous population of adolescents in residential care with major psychiatric problems. Obviously, future research on these subgroups is needed, but this study is a first step towards a better understanding of the complexity of disruptive behaviour in this population of adolescents in residential care.

## Additional file

**Additional file 1: Table S1.** Differences between the classes in demographic and admission characteristics. **Table S2.** Differences between the classes in psychopathology and substance use. **Table S3.** Differences between the classes in criminal behaviour and **Table S4.** Differences between the classes in life events.

## Footnotes

1 For reasons of brevity, the term ‘adolescent’ is used throughout the text to include young adults who were sentenced under the Dutch juvenile justice system.
