# Supplementary material for: Many, more, most: four risk profiles of adolescents in residential care with major psychiatric problems
Source: Child Adolesc Psychiatry Ment Health. 2017 Dec 19;11:63. doi: 10.1186/s13034-017-0204-1 (PMC5738157; doi:10.1186/s13034-017-0204-1)
Supplement: Supplementary file 1 — Additional file 1: Table S1. Differences between the classes in demographic and admission characteristics. Table S2. Differences between the classes in psychopathology and substance use. Table S3. Differences between the classes in criminal behaviour and Table S4. Differences between the classes in life events. [file 13034_2017_204_MOESM1_ESM.pdf]

Table S1 Differences between the classes in demographic and admission characteristics (N = 270)

	Overall mean	Class 1 (*n* = 119)	Class 2 (*n* = 70)	Class 3 (*n* = 49)	Class 4 (*n* = 32)	Wald	*p*	Post hoc
Age at admission, in years	16.9	16.9	17.6	16.8	15.6	24.44	.000	1,2,3 > 4<br>2 > 3
Immigrants (1st or 2nd generation)	34.8%	26.4%	58.3%	21.7%	35.4%	13.70	.003	2 > 1,3
Most recent IQ	93.9	93.2	91.9	97.2	97.1	6.13	.110	-
Earliest age of (outpatient) care	8.6	9.1	9.2	8.0	6.8	8.33	.040	1,2 > 4
Only child	15.4%	11.6%	15.8%	17.5%	25.6%	2.91	.410	-
Judicial measure						16.09	.013	2,3 ≠ 4
Criminal law	47.8%	46.1%	68.9%	46.6%	10.0%			
Civil law	43.7%	45.7%	29.5%	36.1%	79.1%			
Voluntary	8.5%	8.2%	1.7%	17.3%	10.9%			

Table S2 Differences between the classes in psychopathology and substance use (N = 270)

	Overall mean	Class 1 (*n* = 119)	Class 2 (*n* = 70)	Class 3 (*n* = 49)	Class 4 (*n* = 32)	Wald	*p*	Post hoc
Disruptive behaviour disorder	48.9%	54.4%	60.8%	29.8%	32.2%	11.37	.010	1,2 > 3<br>2 > 4
Autism spectrum disorder	42.6%	50.0%	17.4%	74.5%	20.1%	28.64	.000	1,3 > 2,4<br>3 > 1
Attention deficit/hyperactivity disorder	23.3%	28.7%	11.3%	23.3%	29.9%	4.01	.260	-
Substance disorder	22.6%	33.1%	31.7%	0.2%	0.6%	194.67	.000	1,2 > 3,4
Reactive attachment disorder	12.6%	3.0%	23.8%	5.7%	35.0%	15.83	.001	2,4 > 1,3
Schizophrenia or another psychotic disorder	9.3%	11.9%	15.6%	0.1%	0.1%	103.47	.000	1,2 > 3,4
Mood disorder	8.5%	8.9%	8.0%	8.9%	7.6%	.08	.990	-
Anxiety disorder	8.1%	3.6%	8.9%	10.1%	20.7%	7.38	.061	-
Personality disorder	11.3%	5.5%	29.8%	2.0%	11.3%	6.87	.076	-
Mental retardation	5.9%	6.4%	9.9%	3.0%	0.2%	6.16	.100	-
Soft drug use	69.5%	98.0%	89.1%	2.8%	27.1%	49.64	.000	1,2 > 3,4<br>4 > 3
Hard drug use	27.2%	37.0%	44.3%	0.2%	0.7%	214.33	.000	1,2 > 3,4
Alcohol use	72.7%	89.6%	89.3%	31.8%	39.8%	41.83	.000	1,2 > 3,4

Table S3 Differences between the classes in criminal behaviour (N = 270)

	Overall mean	Class 1 (*n* = 119)	Class 2 (*n* = 70)	Class 3 (*n* = 49)	Class 4 (*n* = 32)	Wald	*p*	Post hoc
No conviction	18.9%	13.4%	10.5%	21.9%	53.1%	18.03	.000	4 > 1,2,3
Drug offence	4.4%	7.5%	2.6%	1.7%	1.3%	2.65	.450	-
Vandalism (property)	30.7%	40.7%	40.6%	10.3%	3.8%	12.59	.006	1,2 > 3,4
Property offence without violence	45.2%	52.7%	67.6%	9.6%	23.7%	22.69	.000	1,2 > 3,4
Moderate violent offence	50.0%	55.8%	62.7%	36.3%	21.9%	12.25	.007	1,2 > 4<br>2 > 3
Violent property offence	19.6%	25.4%	29.6%	4.7%	0.3%	48.21	.000	1,2 > 3,4<br>3 > 4
Serious violent offence	7.8%	11.7%	7.0%	4.4%	0.2%	13.52	.004	1,2,3 > 4
Sex offence	13.3%	2.0%	12.9%	44.1%	9.1%	21.37	.000	3 > 1,2,4
Manslaughter	3.3%	0.1%	5.0%	4.2%	10.7%	2.61	.460	-
Arson	0.7%	0.6%	1.8%	0%	0.1%	10.62	.014	1,2 > 3
Murder	2.6%	1.0%	5.8%	3.7%	0%	12.10	.007	2,3 > 4
Age first criminal behaviour	13.8	14.2	13.2	13.9	13.8	3.49	.320	-
Age first violent behaviour	14.4	14.8	14.1	14.7	12.9	5.53	.140	-

Table S4 Differences between the classes in life events (N = 270)

	Overall mean	Class 1 (*n* = 119)	Class 2 (*n* = 70)	Class 3 (*n* = 49)	Class 4 (*n* = 32)	Wald	*P*	Post hoc
Chronic illness or hospitalization	15.0%	15.5%	20.5%	8.6%	11.2%	2.43	.490	-
Victim physical abuse (outside the family)	12.7%	8.4%	19.9%	9.6%	18.3%	4.20	.240	-
Victim sexual abuse	8.3%	2.9%	17.4%	8.0%	9.5%	4.52	.210	-
Victim discrimination	2.7%	3.7%	4.0%	0.1%	0%	8.44	.038	1,2 > 4
Financial problems	18.1%	18.5%	36.1%	2.7%	2.6%	10.42	.015	2 > 1,3
Migration	17.1%	12.3%	29.9%	13.1%	13.2%	7.38	.061	-
Fled from another country	3.7%	6.8%	2.8%	0.1%	0.1%	15.68	.001	1,2 > 3<br>1 > 4
Out-of-home-placement	46.5%	42.6%	48.3%	30.2%	82.4%	11.42	.010	4 > 1,2,3
Chronic illness or hospitalization of parents	21.7%	14.0%	27.0%	28.4%	28.1%	4.46	.220	-
Chronic illness or hospitalization of brother/sister	3.1%	5.8%	1.1%	1.8%	0%	14.73	.002	1,3 > 4
Drug abuse parents	31.2%	9.2%	70.8%	7.7%	61.3%	41.56	.000	2,4 > 1,3
Psychopathology parents	39.9%	26.7%	60.1%	36.2%	51.3%	11.41	.010	2 > 1,3
Psychopathology brother/sister	19.0%	15.1%	29.0%	13.4%	22.0%	3.59	.310	-
Divorced parents	64.3%	47.5%	87.7%	58.1%	86.0%	20.13	.000	2,4 > 1,3
Problems with new parent(s)	19.0%	14.7%	32.9%	3.4%	29.4%	10.39	.016	2,4 > 3<br>2 > 1
Financial problems parents	18.3%	5.7%	36.9%	13.3%	33.9%	14.70	.002	2,4 > 1<br>2 > 3
Work problems or unemployment parents	25.0%	21.7%	29.2%	20.8%	35.0%	2.26	.520	-
Deceased parents	9.5%	8.3%	13.8%	7.0%	9.3%	1.39	.710	-
Deceased brother/sister	3.4%	4.1%	4.7%	0%	3.4%	22.92	.000	1,2,4 > 3
Victim bullying	49.2%	40.6%	50.3%	86.1%	19.5%	18.10	.000	3 > 1,2,4<br>2 > 4
Impregnated a girl	1.9%	0.3%	2.2%	0%	10.2%	19.03	.000	2,4 > 3
